# Pure electrical, highly-efficient and sidelobe free coherent Raman spectroscopy using acousto-optics tunable filter (AOTF)

**DOI:** 10.1038/srep20017

**Published:** 2016-02-01

**Authors:** Zhaokai Meng, Georgi I. Petrov, Vladislav V. Yakovlev

**Affiliations:** 1 Texas A&M University, College Station, TX, 77843, United States.

## Abstract

Fast and sensitive Raman spectroscopy measurements are imperative for a large number of applications in biomedical imaging, remote sensing and material characterization. Stimulated Raman spectroscopy offers a substantial improvement in the signal-to-noise ratio but is often limited to a discrete number of wavelengths. In this report, by introducing an electronically-tunable acousto-optical filter as a wavelength selector, a novel approach to a broadband stimulated Raman spectroscopy is demonstrated. The corresponding Raman shift covers the spectral range from 600 cm^−1^ to 4500 cm^−1^, sufficient for probing most vibrational Raman transitions. We validated the use of the new instrumentation to both coherent anti-Stokes scattering (CARS) and stimulated Raman scattering (SRS) spectroscopies.

Raman spectroscopies, including spontaneous Raman spectroscopy, coherent anti-Stokes Raman spectroscopy (CARS) and stimulated Raman spectroscopy (SRS), have recently captivated significant attention, as they allow label-free, chemical-selective and sensitive imaging and sensing^1^,^2^. With a dramatically expanding scope of applications, which now cover biomedical imaging (e.g.,^3^,^4^), material science (e.g.,^5^) and remote sensing (e.g.,^6^), there is a growing demand for new instrumentation development, which is capable of providing efficient coherent excitation of Raman spectra. In this report, we present a novel approach to an electronically tunable laser system, which is capable of attaining high fidelity SRS and CARS spectra without any moving parts.

In typical Raman applications, to acquire efficient nonlinear optical interaction, a coherent Raman process (SRS or CARS) is usually implemented. Both SRS and CARS are orders of magnitude more efficient than spontaneous Raman microspectroscopy^1^,^7^, thereby allowing video-rate imaging applications with moderate excitation power (e.g.,^2^,^8^). Moreover, the multi-photon nature of SRS and CARS automatically enhances their spatial resolution in three-dimensional sectioning, an essential feature in biomedical imaging^9^. In common coherent Raman spectroscopies, picosecond pulsed lasers are usually employed, as the spectral bandwidth of such pulses perfectly matches the typical linewidth for a Raman transition (i.e. a few wavenumbers)^3^,^10^,^11^,^12^,^13^. Nevertheless, other approaches, including femtosecond and continuous-wave laser based stimulated Raman spectroscopies, stimulated Raman photoacoustic imaging, spectrally tailored excitation stimulated Raman scattering (STE-SRS) and coherent molecular normal modes generation have also been demonstrated^14^,^15^,^16^,^17^,^18^,^19^,^20^,^21^,^22^,^23^. In most of the case, coherent Raman spectroscopies require two laser sources with independent wavelengths (pump and Stokes). To cover the typical vibrational Raman fingerprint region spanning from 800 to 1800 cm^−1^ 24,25,26, investigators usually pair one broadband and one narrowband lasers^26^,^27^,^28^, or pair two narrowband tunable lasers^29^,^30^. The former approach is advantageous in simultaneously acquiring multiple Raman lines. However, the detectors employed in this approach (usually, CCD cameras) fundamentally limit the detection sensitivity and speed. Alternatively, pairing two narrowband lasers would deliver a stronger signal under the same excitation power. In this framework, a possible but not so common solution is to combine two independent lasers sources^31^,^32^,^33^. Another possible approach is to utilize only one fundamental laser source while employing the down-conversion effect provided by optical parametric generation. Tunable laser pulses can be generated either by externally seeding the parametric generation material^34^,^35^,^36^ or by selecting a portion of the active material’s self-generation emissions^3^,^10^,^29^. For example, in Lefrancois *et al.*’s report, they selected a photonic crystal fiber as the optical amplifier, pumped it with a Yb-doped fiber laser, and seeded it with a tunable cw laser^34^. On the other hand, by utilizing an optical parametric oscillator (OPO), Ganikhanov *et al.* performed a wavelength selection procedure by changing the physical status of an OPO, including its temperature, output optical filters and the optical path length of the resonance cavity^29^. This strategy has been inherited and improved in later SRS instrumentations^3^,^10^,^37^. Another approach is to employ a broadband laser source (e.g., a 30-nm-wide Yb-fiber laser in Ozeki’s report), and spectrally pick a desired narrowband emission to serve as the tunable source^13^. Typically, in such approaches, a mechanically movable diffractive grating or galvo-mirror was employed to perform the wavelength selection.

The purpose of this article is to introduce and demonstrate another concept for generating picosecond tunable laser source suitable for SRS/CARS applications. In our approach, we adopted an electronically tunable acousto-optical filter for the purpose of selecting emissions with a desired wavelength. Acousto-optic devices have been investigated with growing interest for their potential application as tunable wavelength filters or switches in microspectroscopy imaging systems^38^ and fiber-optic systems^39^,^40^. A typical acousto-optic device consists of a piezo transducer attached to a birefringent crystal. When an RF driving voltage is applied to the piezo transducer, acoustic waves are generated within the crystal, producing a phase grating that can diffract part of the incident light beam under phase-matching conditions. When tuning the RF driving frequency of the piezo transducer, the diffraction window of the crystal will be changed according to the phase matching condition^41^,^42^. Compared with other wavelength tuning techniques (e.g.,^13^,^29^,^34^), acousto-optical tunable filters (AOTF) provide a pure electrical solution that eliminates all the moving parts (e.g., diffractive gratings) within the system.

In this report, by utilizing an AOTF, we introduce a simple but highly efficient approach suitable for stimulated Raman spectroscopies. Specifically, we adopted a picosecond 1064 nm laser as the only laser source. A red-shifted supercontinuum emission was derived by sending portions of the 1064 nm laser into a single mode fiber. An AOTF was utilized for preparing a tunable seed. The seed was then sent to an optical parametric amplifier (OPA), which was pumped by 532 nm laser doubled from the 1064 nm fundamental emission. The OPA’s output, including the amplified “signal” and “idler”, were selectively combined with another portion of the 1064 nm laser, and then sent to the sample. In this way, a coherent Raman process could be excited, and both stimulated Raman gain/loss and anti-Stokes photons could be recorded.

## Methods

**Figure 1:**
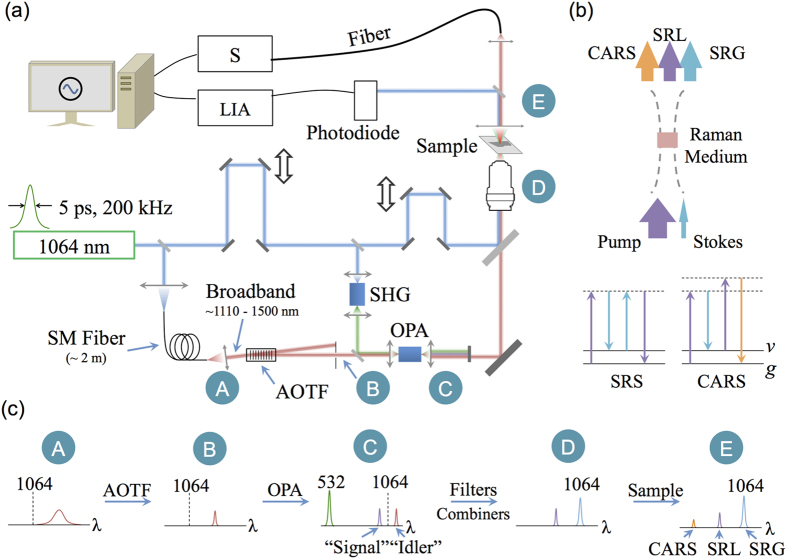
(**a**) Schematics of the experimental setup. SHG: Second Harmonic Generation; OPA: Optical Parametric Generation; AOTF: Acousto-Optical Tunable Filter; S: Spectrometer; LIA: Lock-In Amplifier; (**b**) Illustration of energy transfer in SRS and CARS effects. SRG: stimulated Raman gain; SRL: stimulated Raman loss; *ν*: vibrational state; *g*: ground state; (**c**) The spectrum in each stage on the optical beam path.

Figure 1(a) portrays the basic experimental setup. For all the experiments, we used a home-built picosecond Nd:YVO_4_ laser described in previous report^43^. In this particular application, the repetition rate was tuned to 200 kHz, and the pulse duration was ~ 5 ps. The energy contained in each pulse was greater than 10 μJ. The energy in fundamental wavelength (1064.20 nm) was split into three parts. The first part was sent to a single-mode fiber (~2 meters) for generating a red-shifted supercontinuum emission (~1110-1500 nm). An AOTF (Model: TF1650-1100-2-3-GH40, Gooch & Housego Inc.) was inserted in the beam path of the collimated fiber output for the purpose of inducing diffraction, which could be controlled by the driving frequency. In this way, only a narrowband of the supercontinuum could pass the mechanical iris after the AOTF. The selected narrowband emission was sent to an optical parametric amplifier (OPA, a heated 20-mm LiB_3_O_5_, LBO), and would be served as seed photons (at idler’s side).

The second part of 1064 nm pulses was sent to generate the second-harmonic radiation (532.10 nm) and worked as the pump source of the OPA. Due to the nature of the parametric generation, photons with new wavelength (signal), which is shorter than 1064 nm, will emerge. The underlying physical principle ensures the frequencies of the signal, idler and the pump to follow the relationship: *ω*_signal_ + *ω*_idler_ = *ω*_pump_. Both the amplified signal and idler emissions were in a pulsed manner, and their pulse durations matched the 532 nm pump (i.e., the 1064 nm fundamental laser).

The third part of 1064 nm pulses was combined with the output of the OPA (signal, or idler). The combined beam was focused onto the sample by a spherical lens (N.A. = 0.5). The transmitted photons, including the pump, Stokes, and CARS components, were collected by another spherical lens. The output was analyzed by both a photodiode (Model: 2031, Newport Inc.) and a spectrometer (Shamrock, Andor Technology Inc.). Here, we employed the photodiode to receive 1064 nm emissions (stimulated Raman gain), and used the spectrometer to record the strength of the CARS signal.

To record a Raman spectrum over a broad range, we scanned the AOTF driving frequency and tuned the OPA temperature in all the tests. When detecting the 1064 nm output for SRS, we enabled the optional modulation function of the AOTF and synchronized the lock-in amplifier (SR810, Stanford Research Systems) connected with the photodiode detector. When recording CARS signal, the spectrometer was running in an accumulation mode so that the CARS signal could be completely recorded by the end of the sweeping procedure.

Figure 1(b) illustrates the energy transfer process and the energy diagrams for SRS/CARS process. In these processes, a pump beam at frequency ω_p_ and a Stokes beam at frequency ω_S_ interact with a Raman medium via a four-wave mixing process. When the beat frequency ω_p_ – ω_S_ matches a molecular vibration frequency within the medium, the resonant oscillators will be coherently driven by the external fields, thereby generating a strong anti-Stokes signal at ω_AS_ = 2ω_p_ – ω_S_ (CARS) and inducing an energy transfer between the pump and the Stokes (SRS).

Figure 1(c) explains the spectra at each stage. Point “A” shows the broadband supercontinuum output of the fiber, ranging from ~ 1110 nm to 1500 nm. Point “B” is the output of the AOTF, which worked as a tunable band-pass filter. Point “C” shows the output of the OPA, including the remaining 532 nm pump, the amplified “signal” and “idler”. Point “D” is the spectrum prior to the objective. Only a portion of the OPA output is selected and combined with the 1064 nm pulses. In this particular case, a combination of the amplified “signal” and the 1064 nm pulses is shown. In this way, after the Raman scattering process, the 1064 nm laser will experience a stimulated Raman gain (SRG), the “signal” pulse would experience a stimulated Raman loss (SRL), and the anti-Stokes peaks would emerge as well (see the spectrum illustration for point “E”).

## Results

### AOTF enables sufficient tunability for the light source preparation system

**Figure 2:**
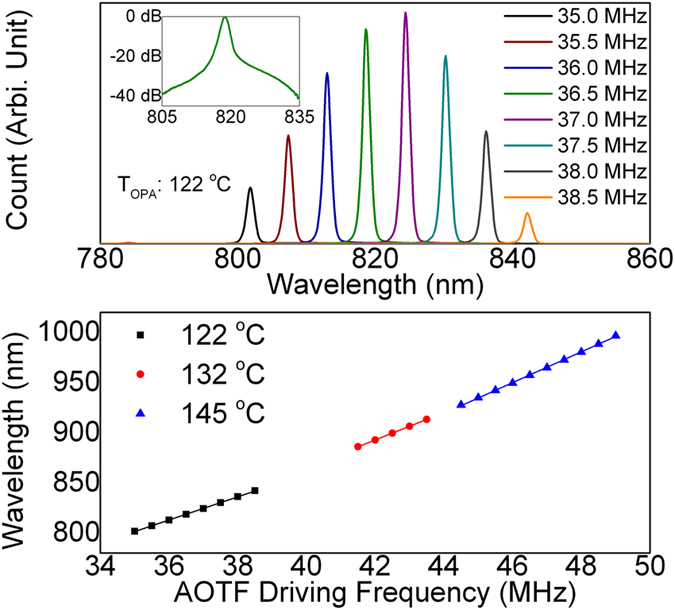
Typical output of the OPA as a function of AOTF driving frequency. Upper: spectrometer reading under different AOTF driving frequency. The OPA temperature was tuned to 122 °C. Lower: The correspondence between AOTF driving frequency and output wavelength. If paired with 1064 nm pump, the entire vibrational Raman range (400–4500 cm^−1^) could be covered.

Figure 2 demonstrates the amplified output of the OPA. When taking the data, the OPA was heated to 122 °C so that its amplification window ranges from 790 to 850 nm. In the data shown in Fig. 2 (upper), we swept the AOTF driving frequency from 35 MHz to 38.5 MHz. The corresponding “idler” seed would, therefore, range from 1400 nm to 1550 nm. Here we only showed the “signal” pulses of the OPA output. Based on the spectrometer readings, the typical linewidth of the OPA output was ~1 nm (14 cm^−1^). The actual linewidth may be narrower, as the spectrometer results are usually broadened due to the blooming effect of CCD cameras. The inset plots the CCD reading in logarithmic scale for 36.5 MHz in AOTF driving frequency. The bottom part of Fig. 2 shows the correspondence between the AOTF driving frequency and the “signal” wavelength. The OPA was heated to three different temperatures. Under each temperature, the correspondence exhibited a linear relationship. In practice, the frequency resolution of the AOTF driver could approach 10 kHz. Therefore, the minimal step size of the OPA output is ~0.05 nm or 0.7 cm^−1^.

### Stimulated Raman process is demonstrated under AOTF-based instrumentation

**Figure 3:**
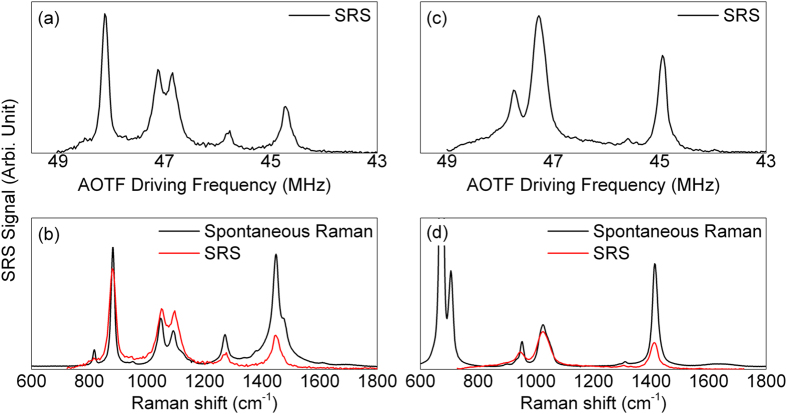
The stimulated Raman signal detected via AOTF setup for (**a**) ethanol and (**c**) an unknown sample (mainly collagen monomers); The comparisons between SRS signal and spontaneous Raman spectrum were shown in (**b**,**d**). The relationship between AOTF driving frequency and Raman shift was calibrated using the data shown in Fig. 2.

Figure 3 shows the presence of the stimulated Raman process. In this demonstration, we chose both ethanol and an unknown liquid (mainly, collagen monomers) as the sample. For 1064 nm laser, the input power was ~5 mW. For the tunable OPA output, the input power ranged from 1 – 50 mW, depending on the wavelength. The parametric crystal’s temperature was set to 145 °C in order to cover Raman shift ranging from 600 cm^−1^ to 1800 cm^−1^. The modulation frequency was ~40 kHz. The integration time was set to 30 ms for the lock-in amplifier. The AOTF driving frequency was swept from 43 MHz to 49 MHz. Figure 3(a,c) show the raw data for these measurements. When setting the photodiode as high-gain, the background level was around 2 μV while the typical peak level could reach 1 mV. The corresponding signal-to-noise ratio (SNR) was ~ 800 in maximum.

Figure 3(b,d) depict the comparisons between the spontaneous Raman and the SRS spectra. The main features were similar between the spontaneous and SRS spectra. However, the SRS spectra were weighted by the amplification efficiency of the OPA. This problem could be overcome by tuning the OPA’s heating temperature. The leaking of the optical filters also induced some background, mainly centered at ~ 1000 cm^−1^ in these tests.

### Coherent anti-Stokes scattering process can be recorded using spectrometers

**Figure 4:**
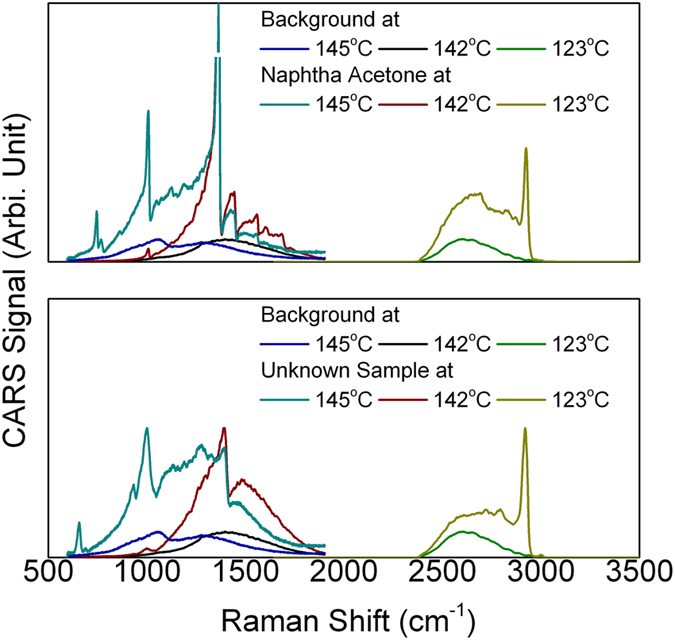
CARS spectrum for acetone (upper) and the unknown sample (lower). The OPA was heated to different temperature. 3000 cm^−1^ Raman lines become visible when the OPA temperature was lowered to 123 °C.

Figure 4 shows the CARS applications using the same optical setup. Here we combined the amplified idler (1110 – 1500 nm) with the 1064 nm pulses. The CARS signal would be located at ~ 800 – 1000 nm. The power of the CARS emission ranges from several microwatts to several hundreds of microwatts. Neutral density filters were applied to avoid saturation. The background was acquired from a glass slide. The dip at 145 °C was induced by the Raman line at ~ 1200 cm^−1^ of the BK-7 glass.

## Discussion

Microscopic optical imaging with a contrast provided by vibrational spectroscopy is important for biomedical researches and material sciences. Various methods and techniques have been proposed and used extensively to prepare laser sources suitable for stimulated Raman spectroscopies. In this study, by combining an AOTF and a single-pass OPA, we have demonstrated yet another approach. Unlike the cw-seeded OPAs, the energy per seeding pulse in this experiment could reach ~0.05 nJ. Considering the pulse duration of the seed, this instantaneous energy level is equivalent with a 10 W cw seeding laser, which is over 100 times stronger than any widely used tunable cw laser in Raman spectroscopies. Therefore, by appropriately overlapping the seed and the pump, OPA could efficiently generate “signal” and “idler” emissions without emitting significant sidelobes. Meanwhile, any moving parts associated with regular tunable cw lasers or OPOs, including gratings and motors, could be eliminated from this system. The associated biomedical imaging applications are on-going and will be reported elsewhere.

In our current instrumentation, the spurious background level (1-2 μV) still limits its applicability. Some nonlinear optical processes, including nonlinear absorption^44^, cross phase modulation and thermal lensing^45^,^46^, would induce output beam intensity variations that may be misinterpreted as an SRS signal. The simplest way to overcome these difficulties is to employ a condenser with greater numerical aperture^10^. These spurious backgrounds can also be manually subtracted by comparing the signal levels in the resonant and non-resonant regions^47^. Alternatively, the undesired nonlinear optical processes can be suppressed by a three-color, double-modulation scheme (stimulated Raman gain and opposite loss detection, SRGOLD)^48^. Our AOTF allows its output to be separately controlled by different channels, making it possible to accommodate the SRGOLD scheme.

Another area of potential improvement in our experimental design is to increase the SNR level. Currently the SNR level of ~500 could lead to some issues in practical applications such as biomedical imaging where weaker signals are expected. The noise was predominantly generated by the unsaturated parametric amplification, which was induced by the relatively short interaction range within the parametric crystal. Small power fluctuation in the seed would result in significant noise in the amplified output. A longer OPA crystal length or a double-pass OPA^49^ would help to suppress the noise, as well as the remaining side-lobes. Correspondingly, the linewidth could also be narrowed, leading to a better spectral resolution^49^.

## Summary

We have demonstrated a simple and efficient approach to preparing laser sources suitable for coherent Raman spectroscopy. This setup consists only one laser source. The entire setup contains no moving parts, making the system laborsaving and cost-effective. We anticipate the wide use of this experimental arrangement in future applications of Raman microspectroscopy.

## Additional Information

**How to cite this article**: Meng, Z. *et al.* Pure electrical, highly-efficient and sidelobe free coherent Raman spectroscopy using acousto-optics tunable filter (AOTF). *Sci. Rep.* **6**, 20017; doi: 10.1038/srep20017 (2016).
